# Soil fertilization affects the abundance and distribution of carbon and nitrogen cycling genes in the maize rhizosphere

**DOI:** 10.1186/s13568-021-01182-z

**Published:** 2021-02-08

**Authors:** Matthew Chekwube Enebe, Olubukola Oluranti Babalola

**Affiliations:** Food Security and Safety Niche Area, Faculty of Natural and Agricultural Sciences, North-West University, Private Bag X2046, Mmabatho, 2735 South Africa

**Keywords:** Soil fertilization, Carbon cycling, Nitrogen cycling, Agriculture, Maize rhizosphere

## Abstract

Soil microbes perform important functions in nitrogen and carbon cycling in the biosphere. Microbial communities in the rhizosphere enhance plants’ health and promote nutrient turnover and cycling in the soil. In this study, we evaluated the effects of soil fertilization with organic and inorganic fertilizers on the abundances and distribution of carbon and nitrogen cycling genes within the rhizosphere of maize plants. Our result showed that maize plants through rhizosphere effects selected and enriched the same functional genes *glnA, gltB*, *gudB* involved in nitrogen cycle as do high compost and low inorganic fertilizer treatments. This observation was significantly different from those of high doses of inorganic fertilizer and low compost manure treated soil. Only alpha amylase encoding genes were selectively enriched by low compost and high inorganic fertilized soil. The other treatments only selected *xynB* (in Cp8), *lacZ* (Cp4), *bglA*, *pldB*, *trpA* (N2), *uidA* (N1) and *glgC*, *vanA* (Cn0) carbon cycling genes in the rhizosphere of maize. Also *Actinomycetales* are selected by high compost, low inorganic fertilizer and control. The control was without any fertilization and the soil was planted with maize. *Bacillales* are also promoted by low compost and high inorganic fertilizer. This indicated that only microbes capable of tolerating the stress of high dose of inorganic fertilizer will thrive under such condition. Therefore, soil fertilization lowers nitrogen gas emission as seen with the high abundance of nitrogen assimilation genes or microbial anabolic genes, but increases carbon dioxide evolution in the agricultural soil by promoting the abundance of catabolic genes involve in carbon cycling.

## Key points

- Soil fertilization has selective effects on the microbial nitrogen cycling genes.
- Fertilization enriched microbial carbon degradation genes in the rhizosphere.
- *Bacillales* a stress tolerant group of microbes, thrives well in the presence of high dose of inorganic fertilizer.

## Introduction

The need to profile microbial community structure and functions are important for the attainment of a sustainable agriculture because of the roles they play in facilitating the biogeochemical cycling of nutrients such as carbon, phosphorus, nitrogen, sulphur and metals (Falkowski et al. 2008). Soil microbes are genetically diverse and plants exert influence on them. These influence varies from plant to plant and is achieved through roots exudation and modifications of soil environmental conditions (water, minerals and temperature) (Denef et al. 2009; Dini-Andreote and van Elsas 2013). The rhizosphere microbial communities can be altered by the specific genotype of the plants growing in the soil (Aira et al. 2010; Lawal and Babalola 2014), which may support microbial biomass formation and metabolic activities that will be inherent in the soil. These interactions at the rhizosphere generally control important biogeochemical cycling involved in carbon cycle, emission of greenhouse gases and cycling of other nutrients.

However, fertilization process and methods have been implicated in altering and shaping the microbial communities and their biological functions in the soil. Studies examining the metabolic potentials of microbiomes present in the rhizosphere of crops like grapevine, soybean, wheat and cucumber, barley (Bulgarelli et al. 2015; Mendes et al. 2014; Ofek-Lalzar et al. 2014; Zarraonaindia et al. 2015) have revealed that a consortium of genes involved in chemotaxis, stress tolerance, nutrient cycling, and growth promotion of plants are in abundance in the rhizosphere. Until now, the separation of plants’ rhizosphere effects (maize) from fertilization on the enrichment of functional genes in the soil is still unclear. However, little is known concerning whether fertilizers have greater effects on microbial genes abundance and activities in the soil or is it the plants that shapes and decides which genes should be expressed more in a fertilized soil, and if so, how does it influence nutrient cycling in the agricultural soil.

Meanwhile, inorganic fertilizer application to the farm drastically affect plants nutrient uptake, increase greenhouse gases emission and eutrophication. This necessitate the need for the use of organic fertilizer or manure derived from plant materials and animal droppings. Organic manure are not only cost effective but also improves microbial biomass formation and activities (Bhattacharyya et al. 2008; Bumunang et al. 2013; Ding et al. 2014; Tejada et al. 2008; Zhu et al. 2016) in the fertilized soils. Organic fertilizers/manure increase the activities and abundance of soil microbes’ more than inorganic fertilizers as reported by Chu et al. (2007) and Zhang et al. (2012).

In this study, we investigated the effects of organic and inorganic fertilizers on the structure and abundance of bacterial genes in the maize rhizosphere. The key objectives of this study are: (i) to evaluate how inorganic and organic fertilizers at different dosages affects the functional bacterial genes (involved in carbon and nitrogen cycling) from maize rhizosphere, and (ii) to determine if maize plants’ rhizosphere effects exert the same functional genes enrichment effects as do the fertilizers used particularly for carbon and nitrogen cycling genes.

## Materials and methods

### Site, samples collection and DNA extraction

**Fig. 1:**
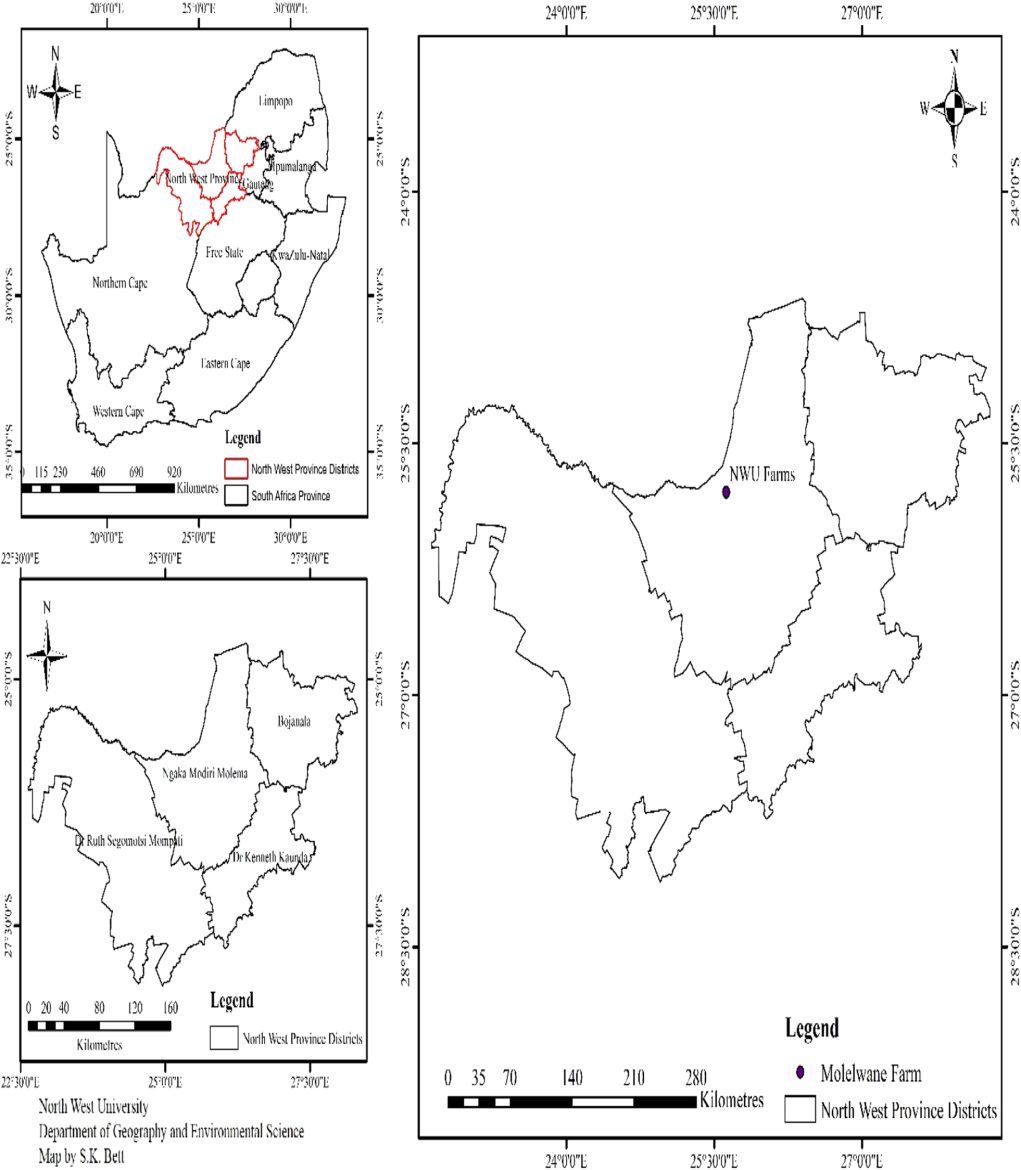
The map of the agricultural sampling site at the Molelwane, North-West University farm, Mafikeng, South Africa

The rhizosphere soil samples were collected from an agricultural site located in Molelwane—a semi-arid savannah climate, near Mafikeng, in the North West Province of South Africa (Fig. 1) (25° 47′ 24.17604″ S, 25° 37′ 9.08328″ E; 25° 47′ 29.97048″ S, 25° 37′ 8.62428″ E; 25° 47′ 23.9604″ S, 25° 37′ 8.43348″ E; 25° 47′ 23.82252′ S, 25° 37′ 8.30064″ E; 25° 47′ 24.11844″ S, 25° 37′ 8.18148″ E; altitude: 1012 m). The soil is a Hutton form, reddish sandy-loam soil with sand (56%), clay (11%), and silt (33%) (Group 1991). The pH is 4.97, total nitrogen 377 mg/kg, phosphorus 10.5 mg/kg, potassium 285 mg/kg, calcium 388 mg/kg, carbon 0.36%, magnesium 162 mg/kg. The farm which was divided into plots were treated with120 kg/ha and 60 kg/ha inorganic NPK (20:7:3) fertilizers and another area with compost manure. The quantity of compost used were 8 tons and 4 tons per hectare (5 treatments × 3 replicates plots per treatment). A total of 20 g of rhizosphere soils were gotten from 7 weeks old maize plants with a 5 cm auger as described by Juma et al. (2018) and Zhang et al. (2013). After soil sampling, the samples collected were put in a sterile plastic bag contained in an ice box and transported to the laboratory for onward analysis in an iceboxes. This was followed by extraction of total community DNA from the collected rhizosphere soil samples with the aid of a PowerSoil DNA isolation kit from MoBio Laboratories, Incorporation in USA) following producer’s instruction.

### Sequencing

DNA (deoxyribonucleic acid) concentrations were examined with the aid of Qubit® dsDNA HS Assay Kit from Life Technologies. And the deoxyribonucleic acid libraries were prepared by the use of Nextera DNA Flex library preparation kit (Illumina Incorporation.) in accordance with the manufacturer’s procedure. A total of 50 nanogram of DNA molecules from each samples were taken for the libraries preparation. This was followed by fragmentation alongside with adapter sequences addition. The adapter molecules were used during PCR cycles together with the addition of unique indices into the samples. The final concentration of the libraries generated were quantified with Qubit® dsDNA HS Assay Kit from Life Technologies. The libraries’ average sizes were determined with the aid of analytical machine—Agilent 2100 Bioanalyzer (from Agilent Technologies). DNA libraries were combined together into an eqimolar ratios of 0.7 nM. The pooled DNA were then sequenced paired end for 300 cycles using the machine—NovaSeq 6000 system (Illumina). This was done at the Mr DNA molecular research laboratory in USA.

### Sequence processing, annotation and statistical analysis

The raw metagenome reads were uploaded into MG-RAST where series of quality control processes were carried out (Meyer et al. 2008). The preprocessing of the reads involved the removal of artificial sequences, host specific sequences and other ambiguous base pairs. This was followed by gene annotation using BLAT algorithm (Kent 2002) and M5NR database (Wilke et al. 2012). The taxonomy and protein coding genes annotation were executed by blasting at M5NR and SEED Subsystem level-function. The BlastX was used for the hit at an e–value cut off of 10E-5, min. alignment length 15 base pairs, and percentage identity of 60. Unannotated sequences were not subjected to any further evaluation/analysis. Also applied was the MG-RAST normalization tool to enable us cut down on the possible experimental error effect from the work. Nitrogen and carbon cycling genes were curated manually from the total gene files gotten from the blasting result of the SEED Subsystem database, level-function. The sequences were used for statistical analysis and the bacterial composition, nitrogen and carbon cycling gene variances were evaluated using one-way ANOVA at p—value less than 0.05. The abundance and distribution of bacterial communities at order level was visualized in bar graphical representation using Microsoft Excel software. An online software—Circos (http://circos.ca/) was employed in plotting the graph of nitrogen cycling genes, while the heatmapper (www.heatmapper.ca/expression/) was used in drawing a heatmap diagram for carbon cycling genes. Evenness, Simpson and Shannon diversity indexes were determined for the rhizosphere samples and contrasted amongst the treatments using Kruskal–Wallis test. The beta diversity was ascertained using PCoA (principal co-ordinate analysis) on the basis of Euclidean distance-matrix and ANOSIM (analysis of similarity) through 9999 permutations. These analysis were carried out on PAST version 3.20 analytical software (Hammer et al. 2001). Principal Co-ordinate Analysis and principal component analysis were performed using the default settings of CANOCO 5v software (numerical data—empty cell are zero and the table representation of compositional data) from Microcomputer Power, Ithaca, New York. The sequences were deposited on NCBI SRA dataset, SRA accession: PRJNA607213.

## Results

### Distribution of Bacteria within the treatments

**Fig. 2:**
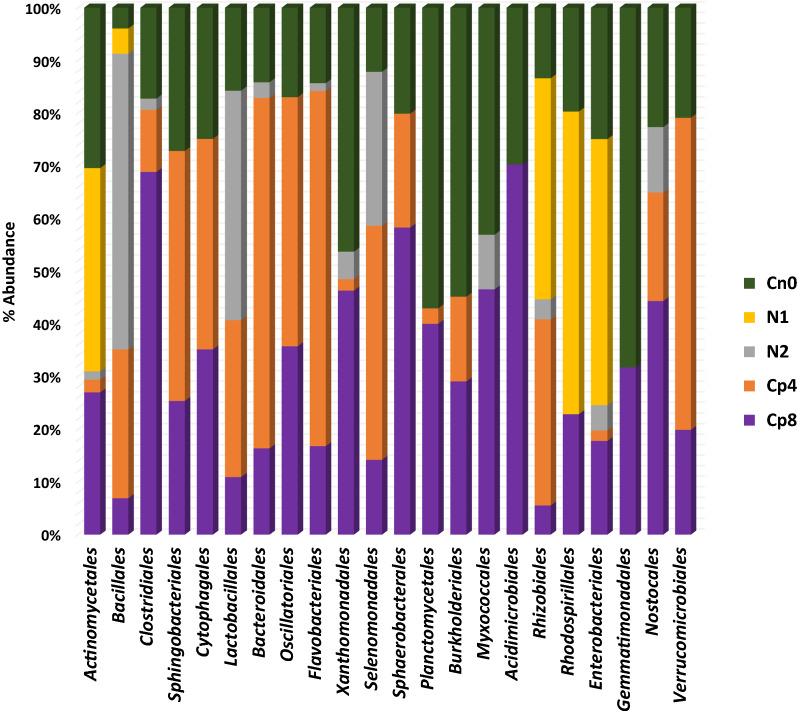
Relative abundance of obvious bacteria order present in the rhizosphere soil treated samples

Analysis of the metagenomes using SEED Subsystem database showed that *Actinomycetales, Bacillales, Sphingobacteriales, Cytophagales, Lactobacillales, Bacteroidales, Flavobacteriales* and *Rhizobiales* were the dominant bacterial order within the fertilized and the unfertilized soil. However, *Bacillales* were the most abundant group in the high dose inorganic (N2) fertilized soil and low compost (Cp4) manure fertilized soil (Fig. 2). Also *Actinomycetales* were most abundant in low dose inorganic (N1) fertilized soil, the control (Cn0) and the high dose compost (Cp8) manure fertilized maize rhizosphere soil. The bacterial order relative abundance is not significant (P = 0.94) among the treated and control soils.

### Nitrogen cycling genes observed within the treated and untreated soils

**Fig. 3:**
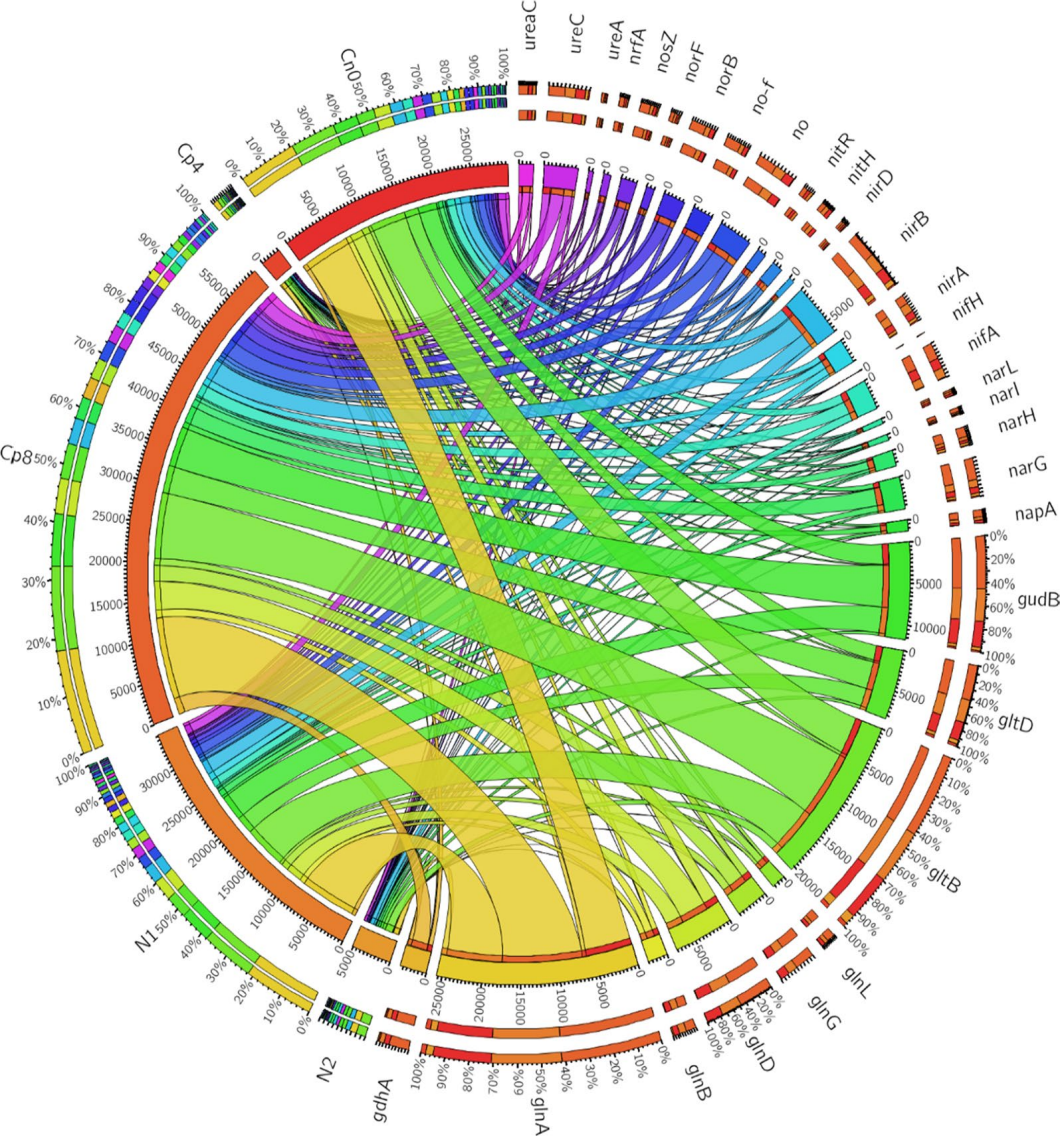
Nitrogen cycling genes found in maize rhizosphere soil under fertilizer treatments plotted with an online software—Circos. The meaning of the gene abbreviations are (*glnA*) glutamine synthetase, (*gltB*) glutamate synthase (NADPH/NADH) large chain, (*gudB*) glutamate dehydrogenase, (*glnD*) uridylyltransferase, (*gltD*) glutamate synthase (NADPH/NADH) small chain, (*nirB*) nitrite reductase (NAD(P)H) large subunit, (*narG*) nitrate reductase 1, alpha subunit, (*gdhA*) glutamate dehydrogenase (NADP+), (*glnG*) two-component system, nitrogen regulation response regulator GlnG, (no) nitrate reductase catalytic subunit [EC:1.7.99.4], (*ureC*) urease subunit alpha, (*nifA*) Nif-specific regulatory protein, (no-F) nitrite reductase (NO-forming), (*norB*) nitric oxide reductase subunit B, (*nosZ*) nitrous-oxide reductase, (*glnB*) nitrogen regulatory protein P-II 1, (*ureaC*) urea carboxylase, (*narH*) nitrate reductase 1, beta subunit, (*nirA*) ferredoxin-nitrite reductase, (*glnL*) two-component system, nitrogen regulation sensor histidine kinase, (*napA*) periplasmic nitrate reductase, (*nrfA*) cytochrome c-552, (*nitH*) nitrile hydratase, (*nitR*) nitrate reductase (NADH), (*norF*) nitric-oxide reductase, (*narI*) nitrate reductase 1, gamma subunit, (urea) urease subunit gamma, (*nirD*) nitrite reductase (NAD(P)H) small subunit, (*narL*) two-component system, nitrate/nitrite response regulator, (*nifH*) nitrogenase iron protein NifH. Note: no, ureaC, nitH, no-f and nitR are our own abbreviation to contain it within the circos plot. These genes have no specific symbol or abbreviations representing them

**Fig. 4:**
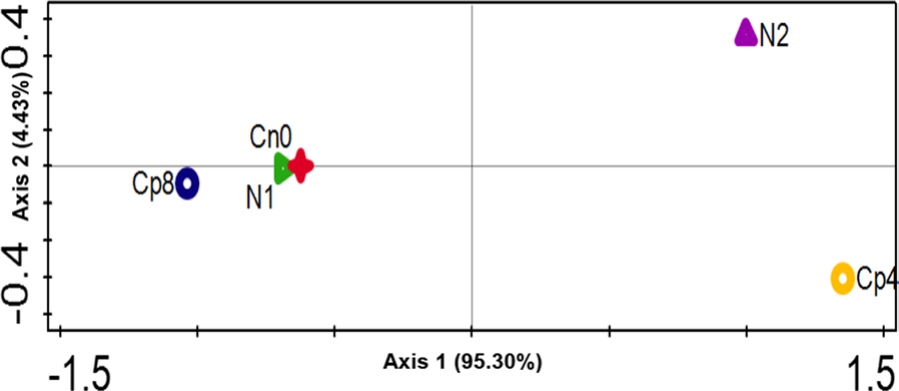
Principal coordinate analysis (PCoA) of the functional nitrogen cycling genes obtained from the fertilized and unfertilized maize rhizosphere soil samples

**Fig. 5:**
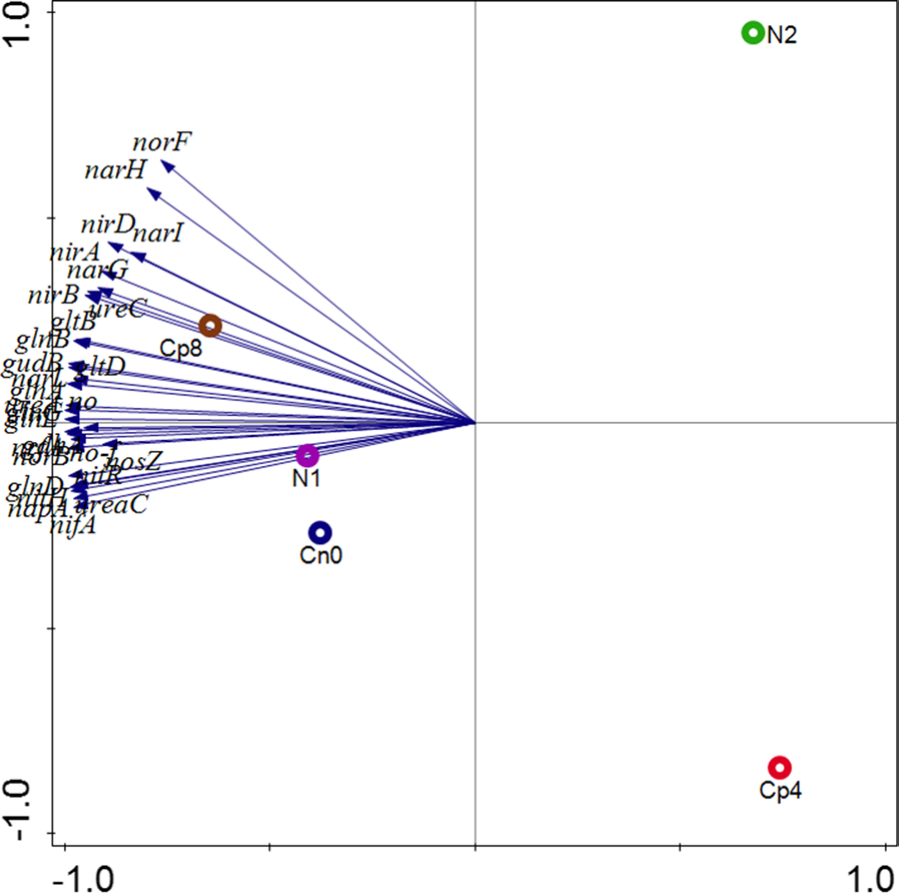
Principal component analysis of the nitrogen cycling genes obtained under fertilization and unfertilized maize rhizosphere soil samples. Axis 1 and Axis 2 explains 94.89% and 3.74% variations. The abbreviated symbols are explained in Fig. 3

Of the 4993 genes found within the samples, 37 of them were the most abundant gene families present in all treatments. These genes (*glnA, gltB, gudB, nifA, nirB, ureC* and others) were involved in nitrogen fixation, nitrification, denitrification, assimilatory nitrogen reduction, dissimilatory nitrogen reduction to ammonium ions, ammonification (Fig. 3). The abundance of these genes differed significantly (p < 0.05) between the treatments and control. Diversity indices-Shannon, Simpson and evenness (Additional file 1: Table S1) were employed to understand the alpha diversity of the nitrogen cycling genes. These diversity indices showed that there were significant differences (Kruskal–Wallis, P-value = 6.87 × 10^–17^) in alpha diversity of the nitrogen cycling genes (Additional file 1: Table S1). Moreover, the analysis of similarity (ANOSIM) indicated that there was a much significant difference (P-value = 0.01 and R-value = 0.55) for the beta diversity, that is, the diversity that exist across the fertilized soils and the control as shown by the principal co-ordinate analysis—PCoA (Fig. 4). Also, the Principal component analysis was conducted to represent how the Nitrogen cycling genes were distributed across the treatments and the control samples (Fig. 5).

### Carbon cycling genes under the treatments and control samples

**Fig. 6:**
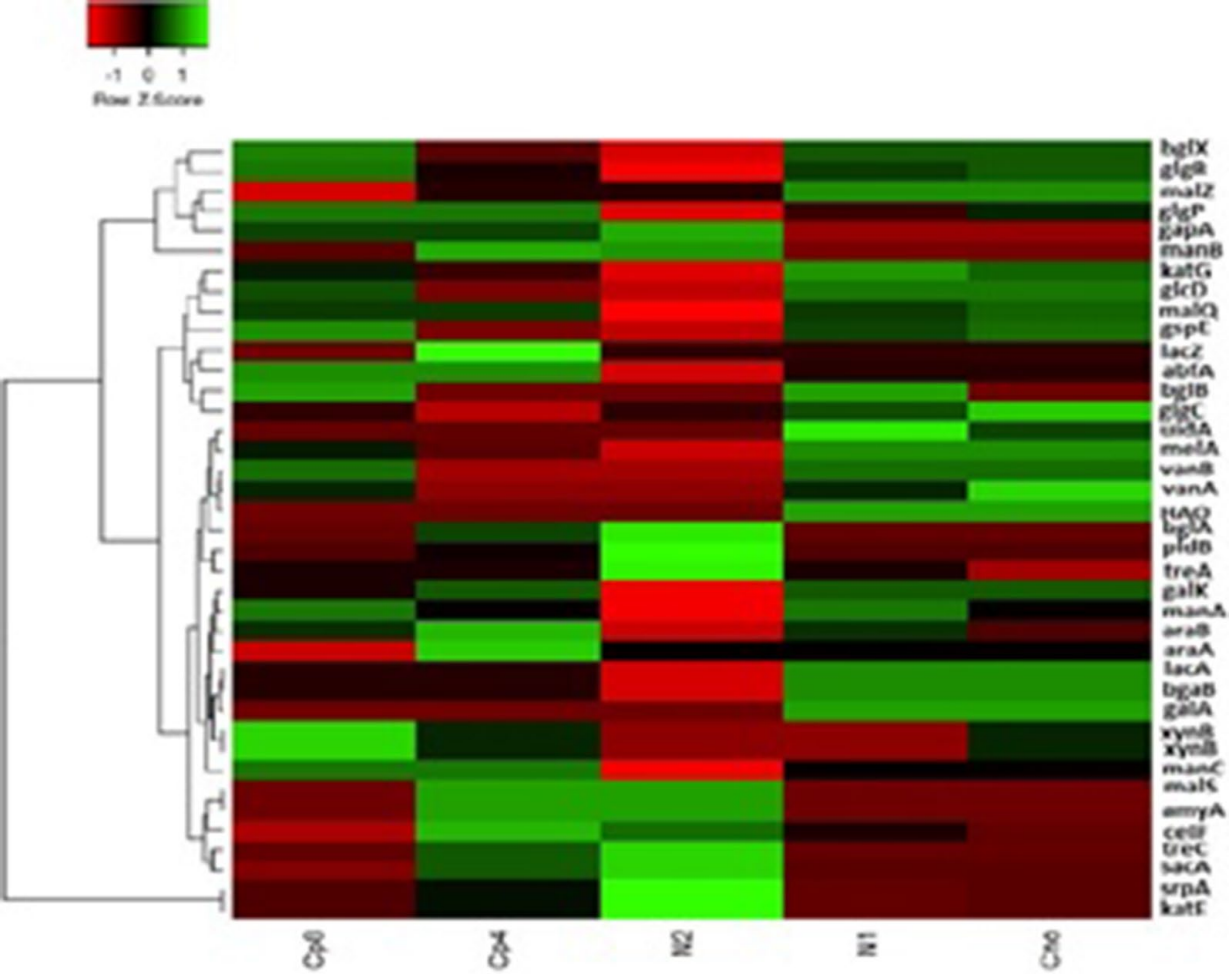
Relative abundance of carbon cycling genes present at maize rhizosphere under fertilization treatments. The abbreviations meaning are (*amyA*) alpha-amylase, (*glgB*) 1,4-alpha-glucan branching enzyme, (*bglX*) beta-glucosidase, (*glgP*) starch phosphorylase, (*manB*) phosphomannomutase, (*gapA*) glyceraldehyde 3-phosphate dehydrogenase, (*malZ*) alpha-glucosidase, (*malQ*) 4-alpha-glucanotransferase, (*gspE*) general secretion pathway protein E, (*katE*) catalase, (*glgC*) glucose-1-phosphate adenylyltransferase, (*bglB*) beta-glucosidase, (*manC*) mannose-1-phosphate guanylyltransferase, (*manA*) mannose-6-phosphate isomerase, (*lacZ*) beta-galactosidase, (*xynB*) xylan 1,4-beta-xylosidase, (*galK*) galactokinase, (*araB*) L-ribulokinase, (*araA*) L-arabinose isomerase, (*lacA*) beta-galactosidase, (*glcD*) glycolate oxidase, (*vanA*) vanillate monooxygenase, (*vanB*) vanillate monooxygenase, (*srpA*) catalase, (HAO) (S)-2-hydroxy-acid oxidase, (*treA*) alpha,alpha-trehalase, (*sacA*) beta-fructofuranosidase, (*treC*) trehalose-6-phosphate hydrolase, (*xynB*) xylan 1,4-beta-xylosidase, (*pldB*) lysophospholipase, (*uidA*) beta-glucuronidase, (*bgaB*) beta-galactosidase, (*abfA*) alpha-N-arabinofuranosidase, (*galA*) alpha-galactosidase, (*melA*) alpha-galactosidase, (*malS*) alpha-amylase, (*bglB*) beta-glucosidase, (*bglA*) 6-phospho-beta-glucosidase, (*celF*) 6-phospho-beta-glucosidase, (*katG*) catalase-peroxidase

**Fig. 7:**
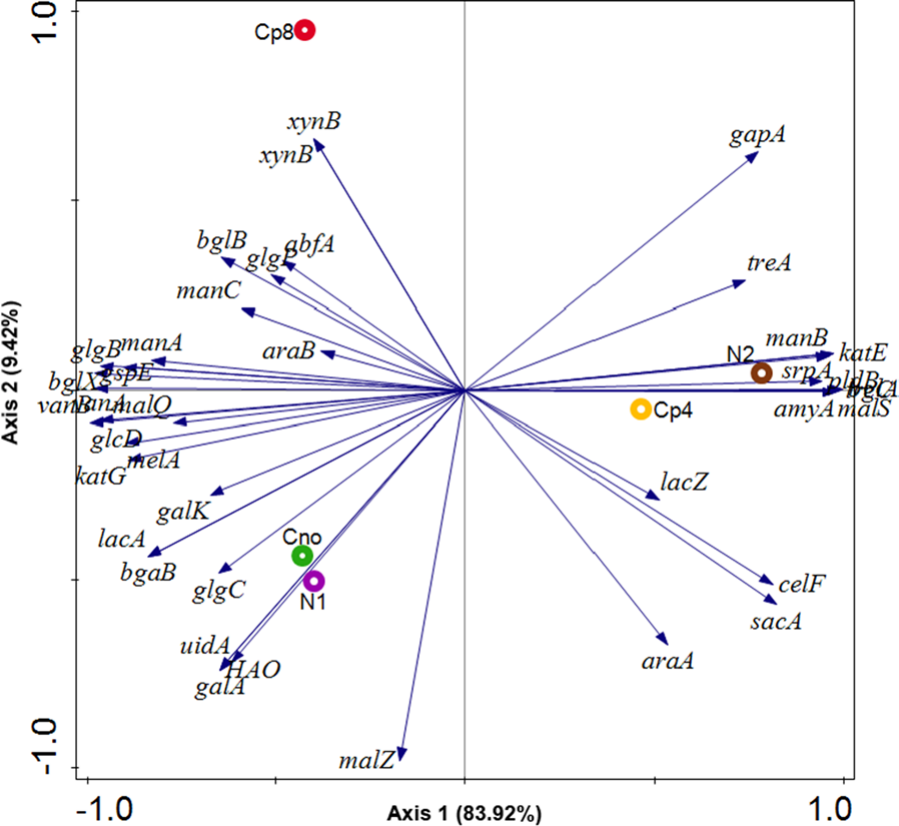
PCA (Principal component analysis) of carbon cycling genes obtained under organic, inorganic and unfertilized rhizosphere soil samples from maize plants

A total of 39 carbon cycling genes were found dominant across all the treatments. They were involved in cellulose, hemicellulose, starch, carbohydrate, cello-oligosaccharides and lignin degradation. None of the carbon fixation genes was found in the treatments and the control rhizosphere soil samples. The abundance of carbon cycling genes differed significantly (P < 0.05) between the samples (Fig. 6). We used Simpson, Shannon and evenness indexes to show the alpha diversity of the carbon cycling genes in the treatments and the control. And they indicated that there was no significant differences (P-value = 0.89) in the carbon cycling genes alpha diversity (Additional file 1: Table S2). There was significant difference as depicted by ANOSIM (P-value = 0.01; R value = 0.55) in beta diversity of the carbon cycling genes and this is in agreement with the Principal coordinate analysis (Additional file 1: Figure S1). Principal Component analysis were equally performed to show the distribution of the carbon cycling genes across the rhizosphere soil samples under the investigation (Fig. 7).

## Discussion

Microbes are the agents that facilitate the transition and transformation of nitrogen and carbon containing compounds from one form to another and soil fertilization significantly affects these microbial communities (Lammel et al. 2015) and their activities in the cycling of these nutrients. Few studies in the past have investigated the impacts of organic compost manure and inorganic fertilizers on the functional genes abundance, but none had considered whether these functional genes abundance are influenced by plants’ rhizosphere effect or the fertilization effects. In this study, we evaluated the abundance of maize rhizosphere bacterial functional genes implicated in nitrogen and carbon cycles under organic and inorganic fertilization. We discovered that the application of high doses of compost manure, low inorganic fertilizer and untreated control had significant impacts on abundance of the bacterial communities and functional genes responsible for nitrogen and carbon cycles than the addition of low quantity of compost manure and high doses of inorganic fertilizer.

The nutrient cycling processes performed by soil-borne microbes like nitrification, denitrification, ammonification, nitrogen fixation, mineralization, carbon degradation, carbon fixation etc. involve series of enzymatic catalysed steps and are performed by a number of interrelated-microbial groups in the soil. The abundance of gene families involved in these processes could give a clue on the rates of biogeochemical cycling of these nutrients under fertilization treatments. In our study, there exist an abundance of gene families involved in nitrogen cycle such as *gln* (glutamine synthetase), *gud* (glutamate dehydrogenase), *nir* (nitrite reductase), *glt* (glutamate synthase), no (nitrate reductase), *ureC* (encoding urease alpha subunit for ammonification) in the maize rhizosphere under fertilized and unfertilized treatments (Fig. 3).

The product of nitrification, which is nitrate (NO_3_^2−^), is the key substrate for denitrification process, therefore, the abundance of the genes implicated in denitrification has significantly contributed to the abundance of nitrogen cycling microbial communities. The nitrite reductase gene families (*nirB*, nitrite reductase (no-f), *nirA*, *nirD*) detected within the samples are responsible for catalyzing the conversion of nitrate to gaseous nitrogen (Butterbach-Bahl et al. 2013; Zumft 1997). They are significantly low in abundance and unaffected by the various treatments applied (Fig. 2). Denitrification is the basic avenue for nitrogen loss in the agricultural soil (Igiehon et al. 2018; Zhu et al. 2018). Other genes involved in denitrification process are *nar*, *nap* encode enzymes that reduces NO_2_^−^ to ammonium ions as well as *nrfA* which serves as marker genes for the identification of microbes that mediate these processes of denitrification (Simon 2002; Welsh et al. 2014). Surprisingly, only a few nitrogen fixing genes (*nifH*) was detected in all the samples. This implies that very few microbial families possess nitrogen fixation genes and are capable of fixing atmospheric nitrogen into the soil. Also non-leguminous plants like maize and the fertilization types adopted could likely support a wide proliferation of microbes bearing these genes as well as their expression in the rhizosphere (Lindstrom and Mousavi 2020; Raymond et al. 2004).

Nitrate reduction is done by nitrate reductase enzymes encoded by *napA*, *narG* and other *nar* gene families (Sánchez and Minamisawa 2018; Vaccaro et al. 2016). Obviously, the quantity of carbon compounds present in the soil (electron donors) in relation to electron acceptors (nitrates) have resultant effects on the emission of N_2_O during denitrification processes (Amoo et al. 2017; Felgate et al. 2012; Richardson et al. 2009) and as observed in Fig. 2, in relation to the treatments at maize growth phase of 7 weeks, the rhizosphere effects of the plants greatly affected the abundance of denitrifying functional genes from the maize rhizosphere. Thus, both organic and inorganic fertilization supported very little emission of nitrogenous gases from the soil. The rhizosphere of maize plants harbors a unique community of nitrifying bacteria as well as possess a rich functional genes that includes *nifH* (nitrogen fixation), *gdh*, *ureC* (for ammonification), *amoA*, *hao* (implicated in nitrification) and *narG*, *nirS, nirK, norB, nosZ* (denitrification) when compared to the bulk soil (Ai et al. 2013; Cheneby et al. 2004; Li et al. 2014; Wang et al. 2017).

On the other hand, genes family involved in ammonia or ammonium assimilation to form glutamine and glutamate like *glnA, gltB, gudB, gltD* are highly abundant in the high dose compost (Cp8), control (Cn0) and low inorganic fertilizer (N1) rhizosphere soil samples. The gene *glnA* encoding enzyme glutamine synthetase which catalyze the biosynthesis of glutamine from glutamate substrate is the most abundant nitrogen cycling genes observed (Fig. 3). Also *gltB* (glutamate synthase) is the second most abundant gene. *gdhA* (glutamate dehydrogenase) is responsible for catalyzing the assimilation of ammonium by 2-oxoglutarate to form glutamate. Therefore, glutamine synthetase, glutamate synthase and glutamate dehydrogenase are the central nitrogen metabolic facilitators for ammonium assimilation in the bacteria cells. Studies have shown that intracellular glutamine concentration serves as an indicator of the quantity of environmentally available nitrogen and are often responsible for microbial growth rate modulation (Ikeda et al. 1996). The quantity of glutamine is usually lower than glutamate at every nitrogen availability but gradually decrease as the external nitrogen source becomes scarce or limited (Ikeda et al. 1996). This is evident with the high level of glutamine synthetase coding genes and glutamate synthase functional genes observed in our studies.

However, soil microbes perform critical roles in organic carbon cycling and its fixation (Liang and Balser 2012). The structure of soil microbial communities regulates both the changes in soil-carbon pool and the pattern of such changes (Billings and Ziegler 2008). Our study revealed that the carbon cycling genes involved in carbon degradation differed significantly under the various fertilization treatments. This cycle, though complex, is a very important metabolic processes in biogeochemical cycling of nutrients (Babalola 2014; Rui et al. 2015). The result showed that the abundance of functional genes in soil bacterial community at the rhizosphere could predict the activities of carbon degrading enzymes at the rhizosphere. This is in agreement with (Trivedi et al. 2016). The relative abundance of carbon cycling genes *xynB* (in Cp8), *lacZ* (Cp4), *bglA*, *pldB*, *trpA* (N2), *uidA* (N1) and *glgC*, *vanA* (Cn0) were significantly different in the observed treatments (Fig. 6), but the functional genes *malS* and *amyA* were most abundant in treatment Cp4 and N2 (Fig. 7). The screened metagenomes showed genes involved in cellulose, carbohydrate, hemi-cellulose, lignin and simple sugars degradation (Fig. 7). Therefore, the fertilization treatments do not have a common carbon cycling genes it promotes rather each treatments select a unique set of carbon degradation genes. To our understanding as observed in our study, neither the fertilization treatments nor the maize plants enrich and support carbon fixation genes rather they promoted carbon degradation ones. This implies that agricultural activities of soil fertilization could contribute to the remarkable production of greenhouse gases—carbon dioxide.

Finally, the relative abundance of bacterial functional marker genes obtained in our study describes the processes peculiar to the biogeochemical cycling of nitrogen and carbon in the soil. This gives a better understanding on how soil fertilization with organic and inorganic fertilizers affects the cycling of these nutrients at the rhizosphere of maize plants. The study showed that low quantity of chemical fertilizer, high dose of compost and no fertilization promoted nitrogen cycling genes particularly those involved in ammonium assimilation. They also aided the selection of only a specific group of carbon degradation genes that are peculiar to each treatment. This suggest that soil fertilization lower nitrogen gas emission but increases carbon dioxide evolution in the agricultural soil. Also *Actinomycetales* are selected by high compost, low inorganic fertilizer and control, while *Bacillales* are promoted by low compost and high inorganic fertilizer. This indicated that only microbes capable of tolerance to the stress of high dose of inorganic fertilizer will thrive under such conditions.

## Supplementary Information

**Additional file 1.** Additional tables and figure.

## Data Availability

The data is deposited at NCBI SRA under the accession number: PRJNA607213.
