# Supplementary material for: Soil fertilization affects the abundance and distribution of carbon and nitrogen cycling genes in the maize rhizosphere
Source: AMB Express. 2021 Feb 8;11:24. doi: 10.1186/s13568-021-01182-z (PMC7870749; doi:10.1186/s13568-021-01182-z)
Supplement: Supplementary file 1 — Additional file 1. Additional tables and figure. [file 13568_2021_1182_MOESM1_ESM.pdf]

Journal name: AMB Express

Soil fertilization affects the abundance and distribution of carbon and nitrogen cycling genes in the maize rhizosphere

*Matthew Chekwube Enebe, Olubukola Oluranti Babalola**

Food Security and Safety Niche Area, Faculty of Natural and Agricultural Sciences, North-West University, Private Bag X2046, Mmabatho 2735, South Africa, Olubukola.Babalola@nwu.ac.za, +27(0)183892568

Table S1. Diversity index for nitrogen cycling genes present in the rhizosphere soil samples

	Cp8	Cp4	N2	N1	Cn0
Simpson_1-D	0.9189	0.9004	0.9017	0.8996	0.9044
Shannon_H	2.885	2.699	2.627	2.744	2.787
Evenness_e^H/S	0.6176	0.5124	0.4771	0.536	0.5598

Table S2. Diversity indices of carbon cycling genes

	Cp8	Cp4	N2	N1	Cn0
Simpson_1-D	0.9546	0.9558	0.9206	0.9569	0.9563
Shannon_H	3.27	3.322	3.007	3.327	3.317
Evenness_e^H/S	0.7307	0.7489	0.5617	0.7332	0.7258

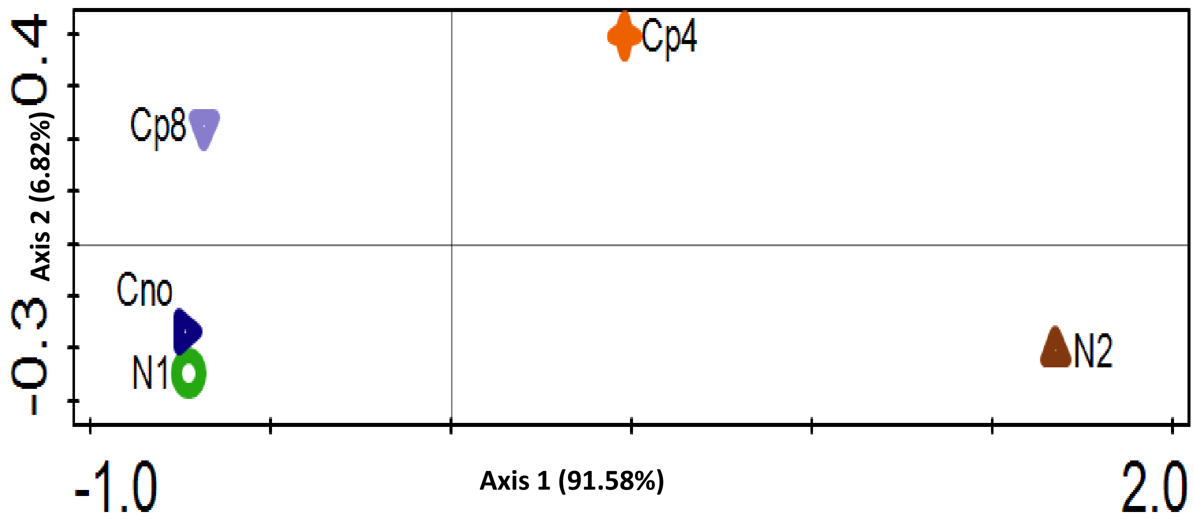

Figure S1. The PCoA for the carbon cycling genes abundance in the maize rhizosphere
